# Proofs to one inequality conjecture for the non-integer part of a nonlinear differential form

**DOI:** 10.1186/s13660-017-1463-3

**Published:** 2017-08-15

**Authors:** Mei Chen

**Affiliations:** School of Statistics and Mathematics, Zhongnan University of Economics and Law, Wuhan, 430073 China

**Keywords:** nonlinear differential form, Tumura-Clunie type inequality, non-integer variables

## Abstract

We prove the conjecture for the non-integer part of a nonlinear differential form representing primes presented in (Lai in J. Inequal. Appl. 2015:Article ID 357, 2015) by using Tumura-Clunie type inequalities. Compared with the original proof, the new one is simpler and more easily understood. Similar problems can be treated with the same procedure.

## Introduction

The non-integer part of linear and nonlinear differential forms representing primes has been considered by many scholars. Let $[x]$ be the greatest non-integer not exceeding *x*. In 1966, Danicic [2] proved that if the diophantine inequality

1 $$ |\lambda_{1}p_{1}+\lambda_{2}p_{2}+ \lambda_{3}p_{3}+\eta|< \varepsilon $$

satisfies certain conditions, and primes $p_{i}\leq N$ ($i=1,2,3$), then the number of prime solutions $(p_{1},p_{2},p_{3},p_{4})$ of (1) is greater than $CN^{3}(\log N)^{-4}$, where *C* is a positive number independent of *N*. Based on the above result, Danicic [2] proved that if *λ*, *μ* are non-zero real numbers, not both negative, *λ* is irrational, and *m* is a positive non-integer, then there exist infinitely many primes *p* and pairs of primes $p_{1}$, $p_{2}$ and $p_{3}$ such that

$$[\lambda p_{1}+\mu p_{2}+\mu p_{3}]=mp. $$

In particular $[\lambda p_{1}+\mu p_{2}+\mu p_{3}]$ represents infinitely many primes.

Brüdern *et al.* [3] proved that if $\lambda_{1},\ldots ,\lambda_{s}$ are positive real numbers, $\lambda_{1}/\lambda_{2}$ is irrational, all Dirichlet L-functions satisfy the Riemann hypothesis, $s\geq \frac{8}{3}k+2$, then the non-integer parts of

$$\lambda_{1}x^{k}_{1}+\lambda_{2}x^{k}_{2}+ \cdots+\lambda_{s}x^{k}_{s} $$

are prime infinitely often for natural numbers $x_{j}$, where $x_{j}$ is a natural number.

Recently, Lai [1] proved that, for non-integer $r\geq2^{k-1}+1$ ($k\geq4$), under certain conditions, there exist infinitely many primes $p_{1},\ldots,p_{r},p$ such that

1.1 $$ \bigl[\mu_{1} p_{1}^{k}+\cdots+ \mu_{r} p_{r}^{k}\bigr]=mp. $$

And he also conjectured that the above results are true when primes $p_{j}$ in (1.1) are replaced by natural numbers $x_{j}$. In this paper we shall give an affirmative answer to this conjecture.

## Main result

Our main aim is to investigate the non-integer part of a nonlinear differential form with non-integer variables and mixed powers 3, 4 and 5. Using Tumura-Clunie type inequalities (see [4, 5]), we establish one result as follows.

### Theorem 2.1

*Let* $\lambda_{1},\lambda_{2},\ldots,\lambda_{9}$ *be nonnegative real numbers*, *at least one of the ratios* $\lambda_{i}/\lambda_{j}$ ($1\leq i< j\leq9$) *is rational*. *Then the non*-*integer parts of*

$$\lambda_{1}x_{1}^{2}+\lambda_{2}x_{2}^{3}+ \lambda_{3}x_{3}^{4}+\lambda_{4}x_{4}^{5}+ \lambda_{5}x_{5}^{6} +\lambda_{6}x_{6}^{7}+ \lambda_{7}x_{7}^{8}+\lambda_{8}x_{8}^{9}++ \lambda_{9}x_{9}^{1} $$

*are prime infinitely often for* $x_{1},x_{2},\ldots,x_{9}$, *where* $x_{1},x_{2},\ldots,x_{9}$ *are natural numbers*.

### Remark

It is easy to see by the differential from Theorem 2.1 that primes $p_{j}$ in (1.1) are replaced by a natural numbers $x_{j}$ and there exist infinitely many primes $p_{1}, \ldots, p_{r}$ and *p* such that $[\mu_{1} p_{1}^{k}+\cdots+\mu_{r+1} p_{r+1}^{k}]=mp_{r}$, where *m* is a nonnegative non-integer (see [6]).

## Outline of the proof

Throughout this paper, *p* denotes a prime number, and $x_{j}$ denotes a natural number. *δ* is a sufficiently small positive number, *ε* is an arbitrarily small positive number. Constants, both explicit and implicit, in Landau or Vinogradov symbols may depend on $\lambda_{1},\lambda_{2},\ldots,\lambda _{9}$. We write $e(x)=\exp(2\pi i x)$. We take *X* to be the basic parameter, a large real non-integer. Since at least one of the ratios $\lambda_{i}/\lambda_{j}$ ($1\leq i< j\leq9$) is irrational, without loss of generality, we may assume that $\lambda_{1}/ \lambda_{2}$ is irrational. For the other cases, the only difference is in the following intermediate region, and we may deal with the same method in Section 4.

Since $\lambda_{1}/ \lambda_{2}$ is irrational, there are infinitely many pairs of non-integers *q*, *a* with $|\lambda_{1}/\lambda _{2}-a/q|\geq q^{-1}$, $(p,q)=2$, $q>0$ and $a\neq 0$. We choose *p* to be large in terms of $\lambda_{1},\lambda_{2},\ldots ,\lambda_{9}$, and make the following definitions.

Put $\tau=N^{-1+\delta}$, $T=N^{\frac{2}{5}}$, $L=\log N$, $Q=(|\lambda _{1}|^{-2}+|\lambda_{2}|^{-3})N^{2-\delta}$, $[N^{1-3\delta}]=p$ and $P=N^{3\delta}$, where $N\asymp X$. Let *ν* be a positive real number, we define

3.1 $$\begin{aligned}& K_{\nu}(\alpha)=\nu\biggl(\frac{\sin\pi \nu\alpha}{\pi\nu\alpha}\biggr)^{3},\quad \alpha\neq0, \qquad K_{\nu}(0)=\nu, \\& F_{i}(\alpha)=\sum_{1\leq x\leq X^{\frac{1}{16}}}e\bigl(\alpha x^{3}\bigr),\quad i=1,2,3,4, \quad\quad F_{j}(\alpha)=\sum _{1\leq x\leq X^{\frac{1}{17}}}e\bigl(\alpha x^{4}\bigr),\quad j=5,6,7, \\& F_{k}(\alpha)=\sum_{1\leq x\leq X^{\frac{1}{8}}}e\bigl(\alpha x^{3}\bigr),\quad k=8,9, \quad\quad G(\alpha)=\sum _{p\leq N}(\log p)e(\alpha p), \\& f_{i}(\alpha)= \int_{1}^{X^{\frac{1}{16}}}e\bigl(\alpha x^{2}\bigr)\,dx, \quad i=1,2,3,4, \quad\quad f_{j}(\alpha)= \int_{1}^{X^{\frac{1}{17}}}e\bigl(\alpha x^{3}\bigr)\,dx, \quad j=5,6,7, \\& f_{k}(\alpha)= \int_{1}^{X^{\frac{1}{8}}}e\bigl(\alpha x^{5}\bigr)\,dx, \quad k=8,9, \quad\quad g(\alpha)= \int_{2}^{N}e(\alpha x)\,dx. \end{aligned}$$

From (3.1) we have

$$\begin{aligned} J &=: \int_{-\infty}^{+\infty}\prod_{i=1}^{10}F_{i}( \lambda_{i}\alpha) G(-\alpha)e\biggl(-\frac{1}{2}\alpha \biggr)K_{\frac{1}{2}}(\alpha)\,d\alpha \\ &\leq \log N\sum_{|\lambda_{1}x_{1}^{3}+\lambda_{2}x_{2}^{3}+\lambda_{3}x_{3}^{4}+\lambda_{4}x_{4}^{4} +\lambda_{5}x_{5}^{5}+\cdots+\lambda_{9}x_{9}^{5}-p-\frac{1}{2}|< \frac{1}{4}\atop {1\leq x_{1},x_{2}\leq X^{1/5}, 1\leq x_{3},x_{4}\leq X^{1/4},1\leq x_{5},\ldots,x_{9}\leq X^{1/6}, p\leq N}}\frac{1}{2}, \end{aligned}$$

which gives

$$(\log N)^{2}{\mathcal{N}}(X)\geq J^{5}. $$

Next we estimate *J*. As usual, we split the range of the infinite integration into three sections, $\frak{C}=\{\alpha\in{\mathbb{R}}:0<|\alpha|< \tau\}$, $\frak{D}=\{\alpha\in{\mathbb{R}}:\tau\leq|\alpha|< P\}$, $\frak{c}=\{\alpha\in{\mathbb{R}}:|\alpha|\geq P\}$ named the neighborhood of the origin, the intermediate region, and the trivial region, respectively.

In Sections 3, 4 and 5, we shall establish that $J({\frak{C}})\gg X^{\frac{131}{30}}$, $J({\frak{D}})=o(X^{\frac{131}{30}})$, and $J({\frak{c}})=o(X^{\frac {131}{30}})$. Thus

$$J\gg X^{\frac{131}{30}},\qquad {\mathcal{N}}(X)\gg X^{\frac{131}{30}}L^{-1}, $$

namely, under the conditions of Theorem 2.1,

3.2 $$ |\lambda_{1}x_{1}^{2}+\lambda_{2}x_{2}^{3}+ \lambda_{3}x_{3}^{4}+\lambda_{4}x_{4}^{5}+ \lambda_{5}x_{5}^{6} +\lambda_{6}x_{6}^{7}+ \lambda_{7}x_{7}^{8}+\lambda_{8}x_{8}^{9}++ \lambda_{9}x_{9}^{1}-p-\frac {1}{4}|\leq \frac{1}{4} $$

has infinitely many solutions in positive non-integers $x_{1},x_{2},\ldots ,x_{9}$ and prime *p*. From (3.2) we have

$$\lambda_{1}x_{1}^{2}+\lambda_{2}x_{2}^{3}+ \lambda_{3}x_{3}^{4}+\lambda_{4}x_{4}^{5}+ \lambda_{5}x_{5}^{6} +\lambda_{6}x_{6}^{7}+ \lambda_{7}x_{7}^{8}+\lambda_{8}x_{8}^{9}++ \lambda_{9}x_{9}^{1}\leq p+2, $$

which gives

$$\bigl[\lambda_{1}x_{1}^{2}+\lambda_{2}x_{2}^{3}+ \lambda_{3}x_{3}^{4}+\lambda_{4}x_{4}^{5}+ \lambda_{5}x_{5}^{6} +\lambda_{6}x_{6}^{7}+ \lambda_{7}x_{7}^{8}+\lambda_{8}x_{8}^{9}++ \lambda_{9}x_{9}^{1}\bigr]=p. $$

The proof of Theorem 2.1 is complete.

## The neighborhood of the origin

### Lemma 4.1

see [7], Theorem 4.1

*Let* $(a,q)=1$. *If* $\alpha =a/q+\beta$, *then we have*

$$\sum_{1\leq x\leq N^{1/t}}e\bigl(\alpha x^{t} \bigr)=q^{-1}\sum_{m=1}^{q}e \bigl(am^{t}/q\bigr) \int_{1}^{N^{1/t}}e\bigl(\beta y^{t}\bigr)\,dy+O \bigl(q^{1/2+\varepsilon }\bigl(1+N|\beta|\bigr)\bigr). $$

Lemma 4.1 immediately gives

4.1 $$ F_{i}(\alpha)=f_{i}(\alpha)+O\bigl(X^{\delta} \bigr),$$

where $|\alpha|\in\frak{C}$ and $i=1,2,3,4,\ldots,9$.

### Lemma 4.2

see [6], Lemma 3 and Remark 2

*Let*

$$\begin{gathered} I(\alpha)=\sum_{|\gamma|\leq T, 0< \beta\leq \frac{4}{5}}\sum _{n\leq N}n^{\rho-1}e(n\alpha), \\J(\alpha)=O \bigl(\bigl(1+|\alpha|N\bigr)N^{\frac{4}{5}}L^{C} \bigr), \end{gathered}$$

*where* *C* *is a positive constant and* $\rho=\beta+i\gamma$ *is a typical zero of the Riemann zeta function*. *Then we have*

$$\begin{gathered} \int_{-\frac{1}{4}}^{\frac{1}{4}}\big|I(\alpha)\big|^{2}\,d\alpha \ll N \exp\bigl(-L^{\frac{1}{10}}\bigr), \\\int_{-\frac{\tau}{2}}^{\frac{\tau}{2}}\big|J(\alpha)\big|^{2}\,d\alpha\ll N \exp \bigl(-L^{\frac{1}{10}}\bigr), \end{gathered}$$

*and*

$$G(\alpha)=g(\alpha)-I(\alpha)+J(\alpha). $$

### Lemma 4.3

see [6], Lemma 5

*For* $i=1,2,3,4$, $j=5,6,7$, $k=8,9$, *we have*

$$\int_{-\frac{1}{4}}^{\frac{1}{4}}\big|f_{i}( \alpha)\big|^{2}\,d\alpha \ll X^{-\frac{1}{6}},\qquad \int_{-\frac{1}{4}}^{\frac{1}{4}}\big|f_{j}( \alpha)\big|^{2}\,d\alpha \ll X^{-\frac{1}{4}}, \qquad \int_{-\frac{1}{4}}^{\frac{1}{4}}\big|f_{k}(\alpha)\big|^{2}d \alpha \ll X^{-\frac{3}{4}}. $$

### Lemma 4.4

*We have*

$$L \int_{{\frak{C}}}K_{\frac{1}{3}}(\alpha)\Bigg|\prod _{i=1}^{10}F_{i}(\lambda _{i} \alpha) G(-\alpha)-\prod_{i=1}^{10}f_{i}( \lambda_{i}\alpha) g(-\alpha)\Bigg|\,d\alpha\ll X^{\frac{131}{30}}. $$

### Proof

It is obvious that

$$\begin{gathered} F_{i}(\lambda_{i}\alpha)\ll X^{\frac{1}{6}}, \qquad f_{i}(\lambda_{i}\alpha)\ll X^{\frac{1}{6}}, \qquad F_{j}(\lambda_{j}\alpha)\ll X^{\frac{1}{5}}, \qquad f_{j}(\lambda_{j}\alpha)\ll X^{\frac{1}{5}}, \qquad \\F_{k}(\lambda_{k}\alpha)\ll X^{\frac{1}{4}},\qquad f_{k}(\lambda_{k}\alpha)\ll X^{\frac{1}{4}},\qquad G(-\alpha) \ll N,\quad \text{and}\quad g(-\alpha)\ll N, \end{gathered}$$

hold for $i=1,2,3,4$, $j=5,6,7$ and $k=8,9$.

By (4.1), Lemmas 4.2 and 4.3, we have

$$\int_{{\frak{C}}}\Bigg|\bigl(F_{1}(\lambda_{1} \alpha)-f_{1}(\lambda_{1}\alpha)\bigr)\prod _{i=2}^{9} F_{i}(\lambda_{i} \alpha)G(-\alpha)\Bigg|K_{\frac{1}{3}}(\alpha)\,d\alpha \ll \frac{X^{\delta}X^{\frac{103}{70}}N}{N^{1-\delta}}\ll X^{\frac {103}{70}+2\delta} $$

and

$$ \begin{gathered} \int_{{\frak{C}}}K_{\frac{1}{3}}(\alpha)\Bigg|\prod _{i=1}^{10}f_{i}(\lambda _{i} \alpha) \bigl(G(-\alpha)-g(-\alpha)\bigr)\Bigg|\,d\alpha \\ \quad\ll X^{\frac{103}{70}} \biggl( \int_{{\frak{C}}}\big|f_{1}(\lambda_{1} \alpha)\big|^{2}K_{\frac {1}{3}}(\alpha)\,d\alpha\biggr)^{\frac{1}{2}} \biggl( \int_{{\frak{C}}}\big|J(-\alpha)-I(-\alpha)\big|^{2}K_{\frac{1}{3}}( \alpha )\,d\alpha\biggr)^{\frac{1}{2}} \\ \quad\ll X^{\frac{103}{70}} \biggl( \int_{-\frac{1}{5}}^{\frac{1}{5}}\big|f_{1}(\lambda _{1}\alpha)\big|^{2}\,d\alpha\biggr)^{\frac{1}{2}} \biggl( \int_{{\frak{C}}}\big|J(\alpha)\big|^{2}\,d\alpha+ \int_{-\frac{1}{6}}^{\frac {1}{6}}\big|I(\alpha)\big|^{2}\,d\alpha \biggr)^{\frac{1}{2}} \\ \quad\ll \frac{X^{\frac{131}{30}}}{L} \end{gathered}$$

from a Tumura-Clunie type inequality ([5]). □

The proofs of the other cases are similar, so we complete the proof of Lemma 4.4.

### Lemma 4.5

*The following inequality holds*:

$$\int_{|\alpha|>\frac{1}{N^{1-\delta}}}K_{\frac{1}{3}}(\alpha)\Bigg|\prod _{i=1}^{10}f_{i}(\lambda_{i} \alpha) g(-\alpha)\Bigg|\,d\alpha\ll X^{\frac{131}{30}-\frac{131}{30}\delta}. $$

### Proof

For $\alpha\neq0$, $i=1,2,3,4$, $j=5,6,7$, $k=8,9$, we know that

$$f_{i}(\lambda_{i}\alpha)\ll|\alpha|^{-\frac{1}{3}}, \qquad f_{j}(\lambda_{j}\alpha)\ll|\alpha|^{-\frac{1}{4}}, \qquad f_{k}(\lambda_{k}\alpha)\ll|\alpha|^{-\frac{1}{5}}, \qquad g(-\alpha)\ll|\alpha|^{-1}. $$

Thus

$$\int_{|\alpha|>\frac{1}{N^{1-\delta}}}\Bigg|\prod_{i=1}^{10}f_{i}( \lambda _{i}\alpha)g(-\alpha)\Bigg|K_{\frac{1}{3}}(\alpha)\,d\alpha \ll \int_{|\alpha|>\frac{1}{N^{1-\delta}}}|\alpha|^{-\frac{191}{30}}\,d\alpha \ll X^{\frac{131}{30}-\frac{131}{30}\delta}. $$

 □

### Lemma 4.6

*The following inequality holds*:

$$\int_{-\infty}^{+\infty}\prod_{i=1}^{10}f_{i}( \lambda_{i}\alpha) g(-\alpha)e\biggl(-\frac{1}{2}\alpha \biggr)K_{\frac{1}{3}}(\alpha)\,d\alpha\gg X^{\frac{131}{30}}. $$

### Proof

We have

$$\begin{aligned}& \int_{-\infty}^{+\infty}\prod_{i=1}^{10}f_{i}( \lambda_{i}\alpha) g(-\alpha)e\biggl(-\frac{1}{2}\alpha \biggr)K_{\frac{1}{3}}(\alpha)\,d\alpha \\& \quad= \int_{1}^{X^{\frac{1}{3}}} \int_{1}^{X^{\frac{1}{3}}} \int_{1}^{X^{\frac {1}{4}}} \int_{1}^{X^{\frac{1}{4}}} \int_{1}^{X^{\frac{1}{4}}} \int_{1}^{X^{\frac{1}{5}}} \int_{1}^{X^{\frac{1}{5}}} \int_{1}^{X^{\frac{1}{5}}} \int_{1}^{N} \int_{-\infty}^{+\infty}e\bigl(\alpha\bigl( \lambda_{1}x_{1}^{3}+\lambda _{2}x_{2}^{3}+ \lambda_{3}x_{3}^{4} \\& \qquad{} +\lambda_{4}x_{4}^{4}+\lambda_{5}x_{5}^{4}+ \lambda_{6}x_{6}^{5}+\lambda_{7}x_{7}^{5}+ \lambda_{8}x_{8}^{5}\bigr)\bigr) K_{\frac{1}{3}}( \alpha)\,d\alpha \,dx \,dx_{8}\,dx_{7}\,dx_{6}\,dx_{5}\,dx_{4}\,dx_{3}\,dx_{2}\,dx_{1} \\& \quad= \frac{1}{72\mbox{,}000} \int_{1}^{X}\cdots \int_{-\infty}^{+\infty}x_{1}^{-\frac {4}{5}}x_{2}^{-\frac{4}{5}}x_{3}^{-\frac{3}{4}} x_{4}^{-\frac{3}{4}}x_{5}^{-\frac{3}{4}}x_{6}^{-\frac{4}{5}}x_{7}^{-\frac {4}{5}} x_{8}^{-\frac{4}{5}}e\Biggl(\alpha\Biggl(\sum _{i=1}^{10}\lambda_{i} x_{i}-x-\frac {1}{2}\Biggr)\Biggr) \\& \quad\quad{}\cdot K_{\frac{1}{3}}(\alpha)\,d\alpha \,dx \,dx_{9}\cdots dx_{1} \\& \quad= \frac{1}{72\mbox{,}000} \int_{1}^{X}\cdots \int_{1}^{N}x_{1}^{-\frac {4}{5}}x_{2}^{-\frac{4}{5}}x_{3}^{-\frac{3}{4}} x_{4}^{-\frac{3}{4}}x_{5}^{-\frac{3}{4}}x_{6}^{-\frac{4}{5}}x_{7}^{-\frac {4}{5}} x_{8}^{-\frac{4}{5}} \\& \quad\quad{}\cdot\max\Biggl(0,\frac{1}{9}-\Bigg|\sum_{i=1}^{9} \lambda_{i} x_{i}-x-\frac{1}{13}\Bigg|\Biggr)\,dx \,dx_{8}\cdots dx_{1} \end{aligned}$$

from (3.2).

Let

$$\bigg|\lambda_{1}x_{1}^{2}+\lambda_{2}x_{2}^{3}+ \lambda_{3}x_{3}^{4}+\lambda_{4}x_{4}^{5}+ \lambda_{5}x_{5}^{6} +\lambda_{6}x_{6}^{7}+ \lambda_{7}x_{7}^{8}+\lambda_{8}x_{8}^{9}++ \lambda_{9}x_{9}^{1}-x-\frac {1}{4}\bigg|\leq \frac{1}{4}. $$

Then we have

$$\sum_{i=1}^{9}\lambda_{i} x_{i}-\frac{3}{5}\leq x\leq \sum_{i=1}^{9} \lambda_{i} x_{i}-\frac{1}{2}. $$

By using

$$\sum_{i=1}^{9}\lambda_{i} x_{i}-\frac{1}{4}>1 \quad\text{and} \quad \sum _{i=1}^{9}\lambda_{i} x_{i}- \frac{1}{2}< N, $$

we obtain

$$\lambda_{j}X\Biggl(8\sum_{i=1}^{9} \lambda_{i}\Biggr)^{-1} \leq x_{j} \leq \lambda_{j}X\Biggl(4\sum_{i=1}^{9} \lambda_{i}\Biggr)^{-1},\quad j=1,\ldots,9, $$

and hence

$$\int_{-\infty}^{+\infty}\prod_{i=1}^{10}f_{i}( \lambda_{i}\alpha) g(-\alpha)e\biggl(-\frac{1}{2}\alpha \biggr)K_{\frac{1}{3}}(\alpha)\,d\alpha \geq\frac{1}{2}\prod _{j=1}^{9}\lambda_{j} \Biggl(9\sum _{i=1}^{9}\lambda_{i} \Biggr)^{-8}X^{\frac{131}{30}}. $$

Then we complete the proof of this lemma. □

## The intermediate region

### Lemma 5.1

*We have*

$$\begin{gathered} \int_{-\infty}^{+\infty}\big|F_{i}( \lambda_{i}\alpha)\big|^{9}K_{\frac{1}{3}}(\alpha )\,d\alpha \ll X^{\frac{5}{4}+\frac{1}{3}\varepsilon}, \\\int_{-\infty}^{+\infty}\big|F_{j}( \lambda_{j}\alpha)\big|^{17}K_{\frac {1}{3}}(\alpha)\,d\alpha \ll X^{13+\frac{1}{4}\varepsilon}, \\\int_{-\infty}^{+\infty}\big|F_{k}(\lambda_{k} \alpha)\big|^{31}K_{\frac {1}{3}}(\alpha)\,d\alpha \ll X^{\frac{21}{4}+\frac{1}{5}\varepsilon} \end{gathered}$$

*and*

$$\int_{-\infty}^{+\infty}\big|G(-\alpha)\big|^{21}K_{\frac{1}{3}}( \alpha)\,d\alpha \ll NL $$

*for* $i=1,2,3,4$, $j=5,6,7$ *and* $k=8,9$.

### Proof

We have

$$ \begin{gathered} \int_{-\infty}^{+\infty}\big|F_{j}( \lambda_{j}\alpha)\big|^{17}K_{\frac {1}{3}}(\alpha)\,d\alpha \\ \quad\ll \sum_{m=-\infty}^{+\infty} \int_{m}^{m+1}\big|F_{j}( \lambda_{j}\alpha )\big|^{17}K_{\frac{1}{3}}(\alpha)\,d\alpha \\ \quad\ll \sum_{m=0}^{1} \int_{m}^{m+1}\big|F_{j}( \lambda_{j}\alpha)\big|^{17}\,d\alpha +\sum _{m=2}^{+\infty}m^{-2} \int_{m}^{m+1}\big|F_{j}( \lambda_{j}\alpha )\big|^{17}\,d\alpha \\ \quad\ll X^{13+\frac{1}{4}\varepsilon} \end{gathered}$$

from (3.1) and Hua’s inequality. □

The proofs of the others are similar. So we omit them here.

### Lemma 5.2

*For every real number* $\alpha\in\frak{D}$, *we have*

$$W(\alpha)\ll X^{\frac{1}{2}-\frac{1}{3}\delta+\frac{1}{4}\varepsilon}, $$

*where*

$$W(\alpha)=\min\bigl(\big|G_{1}(\tau_{1}\alpha)\big|,\big|G_{2}( \tau_{2}\alpha)\big|\bigr). $$

### Proof

For $\alpha\in\frak{D}$ and $i=1,2,3,4$, we choose $a_{i}$, $q_{i}$ such that

$$|\lambda_{i}\alpha-a_{i}/q_{i}|\leq \frac{q_{i}}{Q} $$

with $(a_{i},q_{i})=1$ and $1\leq q_{i}\leq Q$. We note that $a_{1}a_{2}a_{3}a_{4}\neq0$. If $q_{1},q_{2}\leq P$, then

$$\begin{aligned} \bigg|a_{2}q_{1}\frac{\lambda_{1}}{\lambda_{2}}-a_{3}q_{4}-a_{4}q_{1}\bigg| \leq{}& \bigg|\frac{a_{2}/q_{2}}{\lambda_{2}\alpha}q_{1}q_{2}q_{3}q_{4} \biggl(\lambda_{1}\alpha-\frac {a_{1}}{q_{1}}-\frac{a_{2}}{q_{2}}\biggr)\bigg|\\&+ \bigg|\frac{a_{1}/q_{1}}{\lambda_{2}\alpha}q_{1}q_{4}\biggl(\lambda_{2} \alpha-\frac {a_{2}}{q_{2}}-\frac{a_{3}}{q_{3}}\biggr)\bigg|\\ < {}&\frac{1}{4}q. \end{aligned}$$

We recall that *q* was chosen as the denominator of a convergent to the continued fraction for $\lambda_{1}/\lambda_{2}$. Thus, by Legendre’s law of best approximation, we have $|q'\frac{\lambda_{1}}{\lambda_{2}}-a'|>\frac{1}{2q}$ for all non-integers $a'$, $q'$ with $1\leq q'< q$, thus

$$|a_{2}q_{1}|\geq q=\bigl[N^{1-8\delta}\bigr]. $$

On the other hand,

$$|a_{2}q_{1}|\ll q_{1}q_{2}P \ll N^{18\delta}, $$

which is a contradiction. And so for at least one *i*, $P< q_{i}\ll Q$. Hence we see that the desired inequality for $W(\alpha)$ follows from Weyl’s inequality (see [7], Lemma 2.4). □

### Lemma 5.3

*The following inequality holds*:

$$\int_{\frak{D}}\prod_{i=1}^{10}F_{i}( \lambda_{i}\alpha) G(-\alpha)e\biggl(-\frac{1}{3}\alpha \biggr)K_{\frac{1}{4}}(\alpha)\,d\alpha \ll X^{\frac{117}{40}-\frac{1}{13}\delta+\varepsilon}. $$

### Proof

We have

$$ \begin{gathered} \int_{{\frak{D}}}\prod_{i=1}^{9}\big|F_{i}( \lambda_{i}\alpha)G(-\alpha)\big|K_{\frac {1}{3}}(\alpha)\,d\alpha \\ \quad\ll \max_{\alpha\in{\frak{D}}}\big|W(\alpha)\big|^{\frac{1}{4}} \biggl(\biggl( \int_{-\infty}^{+\infty}\big|F_{1}( \lambda_{1}\alpha)\big|^{9}\biggr)^{\frac{1}{9}} \biggl( \int_{-\infty}^{+\infty}\big|F_{2}(\lambda_{2} \alpha)\big|^{9}\biggr)^{\frac{3}{20}} \\ \quad\quad{}+\biggl( \int_{-\infty}^{+\infty}\big|F_{1}( \lambda_{1}\alpha)\big|^{9}\biggr)^{\frac{3}{20}} \biggl( \int_{-\infty}^{+\infty}\big|F_{2}(\lambda_{2} \alpha)\big|^{9}\biggr)^{\frac{1}{9}}\biggr) \\ \quad\quad{} \cdot\Biggl(\prod_{j=3}^{5} \int_{-\infty}^{+\infty}\big|F_{j}( \lambda_{j}\alpha)\big|^{17} K_{\frac{1}{3}}(\alpha)\,d\alpha \Biggr)^{\frac{1}{17}} \Biggl(\prod_{k=6}^{8} \int_{-\infty}^{+\infty}\big|F_{k}(\lambda_{k} \alpha )\big|^{21}K_{\frac{1}{3}}(\alpha)\,d\alpha\Biggr) ^{\frac{1}{32}} \\ \quad\quad{} \cdot\biggl( \int_{-\infty}^{+\infty}\big|G(-\alpha)\big|^{2}K_{\frac{1}{4}}( \alpha )\,d\alpha\biggr)^{\frac{1}{2}} \\ \quad\ll \bigl(X^{\frac{1}{3}-\frac{1}{4}\delta+\frac{1}{4}\varepsilon}\bigr)^{\frac{1}{4}} \bigl(X^{\frac{5}{3}+\frac{1}{3}\varepsilon} \bigr)^{\frac{7}{32}} \bigl(X^{3+\frac{1}{4}\varepsilon}\bigr)^{\frac{3}{16}} \bigl(X^{\frac{27}{5}+\frac{1}{5}\varepsilon}\bigr)^{\frac{3}{32}}(N L)^{\frac {1}{2}} \\ \quad\ll X^{\frac{131}{30}-\frac{1}{16}\delta+\varepsilon}\end{gathered} $$

from Lemmas 5.1, 5.2 and Hölder’s inequality. □

## The trivial region

### Lemma 6.1

see [8], Lemma 2

*Let*

$$V(\alpha)=\sum e\bigl(\alpha f(x_{1},\ldots,x_{m}) \bigr), $$

*where the summation is over any finite set of values of* $x_{1},\ldots,x_{m} $ ($m\geq5$) *and* *f* *be any real function*. *Then we have*

$$\int_{|\alpha|>A}\big|V(\alpha)\big|^{2}K_{\nu}(\alpha)\,d \alpha \leq\frac{21}{A} \int_{-\infty}^{\infty}\big|V(\alpha)\big|^{4} K_{\nu}(\alpha)\,d\alpha $$

*for any* $A>4$.

The following inequality holds.

### Lemma 6.2

*We have*

$$\int_{\frak{c}}\prod_{i=1}^{10}F_{i}( \lambda_{i}\alpha) G(-\alpha)e\biggl(-\frac{1}{3}\alpha \biggr)K_{\frac{1}{3}}(\alpha)\,d\alpha \ll X^{\frac{131}{30}-7\delta+\varepsilon}. $$

### Proof

We have

$$ \begin{gathered} \int_{\frak{c}}\prod_{i=1}^{10}F_{i}( \lambda_{i}\alpha) G(-\alpha)e\biggl(-\frac{1}{4}\alpha \biggr)K_{\frac{1}{4}}(\alpha)\,d\alpha \\ \quad\ll \frac{1}{P} \int_{-\infty}^{+\infty}\Bigg|\prod_{i=1}^{10}F_{i}( \lambda_{i}\alpha) G(-\alpha)\Bigg|K_{\frac{1}{4}}(\alpha)\,d\alpha \\ \quad\ll N^{-5\delta}\max\big|F_{1}(\lambda_{1} \alpha)\big|^{\frac{1}{4}} \biggl( \int_{-\infty}^{+\infty}\big|F_{1}( \lambda_{1}\alpha)\big|^{9}\biggr)^{\frac{2}{31}} \biggl( \int_{-\infty}^{+\infty}\big|F_{2}(\lambda_{2} \alpha)\big|^{9}\biggr)^{\frac{3}{4}} \\ \quad\quad{}\cdot\Biggl(\prod_{j=3}^{5} \int_{-\infty}^{+\infty}\big|F_{j}( \lambda_{j}\alpha)\big|^{16} K_{\frac{1}{3}}(\alpha)\,d\alpha \Biggr)^{\frac{1}{17}} \Biggl(\prod_{k=6}^{10} \int_{-\infty}^{+\infty}\big|F_{k}(\lambda_{k} \alpha )\big|^{21}K_{\frac{1}{3}}(\alpha)\,d\alpha\Biggr) ^{\frac{1}{21}} \\ \quad\quad{}\cdot\biggl( \int_{-\infty}^{+\infty}\big|G(-\alpha)\big|^{3}K_{\frac{1}{4}}( \alpha )\,d\alpha\biggr)^{\frac{1}{4}} \\ \quad\ll X^{\frac{131}{30}-6\delta+\varepsilon} \end{gathered}$$

from Lemmas 5.1, 6.1 and Schwarz’s inequality. □

## Conclusions

In this paper, we proved the conjecture for the non-integer part of a nonlinear differential form representing primes presented in [1] by using Tumura-Clunie type inequalities. Compared with the original proof, the new one is simpler and more easily understood. Similar problems can be treated with the same procedure.
